# The Latvian version of the Juvenile Arthritis Multidimensional Assessment Report (JAMAR)

**DOI:** 10.1007/s00296-018-3959-7

**Published:** 2018-04-07

**Authors:** Ingrida Rumba-Rozenfelde, Ieva Saulite, Ance Rubene, Danute Razuka-Ebela, Marisa Maija-Butnere, Alessandro Consolaro, Francesca Bovis, Nicolino Ruperto, For the Paediatric Rheumatology International Trials Organisation (PRINTO)

**Affiliations:** 1 Faculty of Medicine, University of Latvia, Riga, Latvia; 2 University Children Hospital, Riga, Latvia; 3 Clinica Pediatrica e Reumatologia, Paediatric Rheumatology International Trials Organisation (PRINTO), Istituto Giannina Gaslini, Via Gaslini, 5, 16147 Genoa, Italy; 4 Dipartimento di Pediatria, Università di Genova, Genoa, Italy

**Keywords:** Juvenile idiopathic arthritis, Disease status, Functional ability, Health-related quality of life, JAMAR

## Abstract

The Juvenile Arthritis Multidimensional Assessment Report (JAMAR) is a new parent/patient reported outcome measure that enables a thorough assessment of the disease status in children with juvenile idiopathic arthritis (JIA). We report the results of the cross-cultural adaptation and validation of the parent and patient versions of the JAMAR in the Latvian language. The reading comprehension of the questionnaire was tested in 10 JIA parents and patients. Each participating centre was asked to collect demographic, clinical data and the JAMAR in 100 consecutive JIA patients or all consecutive patients seen in a 6-month period and to administer the JAMAR to 100 healthy children and their parents. The statistical validation phase explored descriptive statistics and the psychometric issues of the JAMAR: the three Likert assumptions, floor/ceiling effects, internal consistency, Cronbach’s alpha, interscale correlations, test–retest reliability, and construct validity (convergent and discriminant validity). A total of 100 JIA patients (2% systemic, 56% oligoarticular, 17% RF negative polyarthritis, 25% other categories) and 204 healthy children, were enrolled at the paediatric rheumatology centre. The JAMAR components discriminated healthy subjects from JIA patients, except for the paediatric rheumatology quality of life (HRQoL), psychosocial health (PsH) subscales, the HRQoL total score and for the school-related problems variable. All JAMAR components revealed good psychometric performances. In conclusion, the Latvian version of the JAMAR is a valid tool for the assessment of children with JIA and is suitable for use both in routine clinical practice and clinical research.

## Introduction

The aim of the present study was to cross-culturally adapt and validate the Latvian parent, child/adult version of the Juvenile Arthritis Multidimensional Assessment Report (JAMAR) [1] in patients with juvenile idiopathic arthritis (JIA). The JAMAR assesses the most relevant parent/patient reported outcomes in JIA, including overall well-being, functional status, health-related quality of life (HRQoL), pain, morning stiffness, disease activity/status/course, articular and extra-articular involvement, drug-related side effects/compliance and satisfaction with illness outcome.

This project was part of a larger multinational study conducted by the Paediatric Rheumatology International Trials Organisation (PRINTO) [2] aimed to evaluate the epidemiology, outcome and treatment of childhood arthritis (EPOCA) in different geographic areas [3].

We report herein the results of the cross-cultural adaptation and validation of the parent and patient versions of the JAMAR in the Latvian language.

## Materials and methods

The methodology employed has been described in detail in the introductory paper of the supplement [4]. In brief, it was a cross-sectional study of JIA children, classified according to the ILAR criteria [5, 6] and enrolled from May 2012 to August 2012. Children were recruited after Ethics Committee approval and consent from at least one parent.

### The JAMAR

The JAMAR [1] includes the following 15 sections:

1. Assessment of physical function (PF) using 15-items in which the ability of the child to perform each task is scored as follows: 0 = without difficulty, 1 = with some difficulty, 2 = with much difficulty, 3 = unable to do and not applicable if it was not possible to answer the question or the patient was unable to perform the task due to their young age or to reasons other than JIA. The total PF score ranges from 0 to 45 and has 3 components: PF-lower limbs (PF -LL); PF-hand and wrist (PF -HW) and PF-upper segment (PF-US) each scoring from 0 to 15 [7]. Higher scores indicating higher degree of disability [8–10].
2. Rating of the intensity of the patient’s pain on a 21-numbered circle visual analogue scale (VAS) [11].
3. Assessment of the presence of joint pain or swelling (present/absent for each joint).
4. Assessment of morning stiffness (present/absent).
5. Assessment of extra-articular symptoms (fever and rash) (present/absent).
6. Rating of the level of disease activity on a 21-circle VAS.
7. Rating of disease status at the time of the visit (categorical scale).
8. Rating of disease course from previous visit (categorical scale).
9. Checklist of the medications the patient is taking (list of choices);
10. Checklist of side effects of medications.
11. Report of difficulties with medication administration (list of items).
12. Report of school/university/work problems caused by the disease (list of items).
13. Assessment of HRQoL, through the physical health (PhH), and psychosocial health (PsH) subscales (5 items each) and a total score. The four-point Likert response, referring to the prior month, are ‘never’ (score = 0), ‘sometimes’ (score = 1), ‘most of the time’ (score = 2) and ‘all the time’ (score = 3). A ‘not assessable’ column was included in the parent version of the questionnaire to designate questions that cannot be answered because of developmental immaturity. The total HRQoL score ranges from 0 to 30, with higher scores indicating worse HRQoL. A separate score for PhH and PsH (range 0–15) can be calculated. [12–14].
14. Rating of the patient’s overall well-being on a 21-numbered circle VAS.
15. A question about satisfaction with the outcome of the illness (Yes/No) [15].

The JAMAR is available in three versions, one for parent proxy-report (child’s age 2–18), one for child self-report, with the suggested age range of 7–18 years, and one for adults.

### Cross-cultural adaptation and validation

The process of cross-cultural adaptation was conducted according to international guidelines with 2–3 forward and backward translations. In those countries for which the translation of JAMAR had been already cross-cultural adapted in a similar language (i.e., Spanish in South American countries), only the probe technique was performed. Reading comprehension and understanding of the translated questionnaires were tested in a probe sample of ten JIA parents and ten patients.

Each participating centre was asked to collect demographic, clinical data and the JAMAR in 100 consecutive JIA patients or all consecutive patients seen in a 6-month period and to administer the JAMAR to 100 healthy children and their parents.

The statistical validation phase explored the descriptive statistics and the psychometric issues [16]. In particular, we evaluated the following validity components: the first Likert assumption [mean and standard deviation (SD) equivalence]; the second Likert assumption or equal items-scale correlations (Pearson r: all items within a scale should contribute equally to the total score); third Likert assumption (item internal consistency or linearity for which each item of a scale should be linearly related to the total score that is 90% of the items should have Pearson *r* ≥ 0.4); floor/ceiling effects (frequency of items at lower and higher extremes of the scales, respectively); internal consistency, measured by the Cronbach’s alpha, interscale correlation (the correlation between two scales should be lower than their reliability coefficients, as measured by Cronbach’s alpha); test–retest reliability or intraclass correlation coefficient (reproducibility of the JAMAR repeated after 1 or 2 weeks); and construct validity in its two components: the convergent or external validity which examines the correlation of the JAMAR subscales with the 6 JIA core set variables, with the addition of the parent assessment of disease activity and pain by the Spearman’s correlation coefficients (*r*) [17] and the discriminant validity, which assesses whether the JAMAR discriminates between the different JIA categories and healthy children [18].

Quantitative data were reported as medians with 1st and 3rd quartiles and categorical data as absolute frequencies and percentages.

The complete Latvian parent and patient versions of the JAMAR are available upon request to PRINTO.

## Results

### Cross-cultural adaptation

The Latvian JAMAR was fully cross-culturally adapted with 2 forward and 2 backward translations with a concordance for 100/123 translations lines (81.3%) for the parent version and 106/120 lines (88.3%) for the child version.

In the probe technique analysis, all the 123 lines of the parent version of the JAMAR were understood by at least 80% of the 10 parents tested (median = 100%; range 90–100%). All the 120 lines of the patient version of the JAMAR were understood by at least 80% of the children (median = 100%; range 90–100%). The text of the parent and patient version of the JAMAR was unmodified after the probe technique.

### Demographic and clinical characteristics of the subjects

A total of 100 JIA patients and 204 healthy children (total of 304 subjects) were enrolled at the paediatric rheumatology centre.

In the 100 JIA subjects, the JIA categories were 2.0% with systemic arthritis, 56.0% with oligoarthritis, 17.0% with RF negative polyarthritis, 13.0% with RF positive polyarthritis, 4.0% with psoriatic arthritis, 7.0% with enthesitis-related arthritis and 1.0% with undifferentiated arthritis (Table 1).

**Table 1:** Descriptive statistics (medians, 1st and 3rd quartiles or absolute frequencies and %) for the 100 JIA patients

	Systemic	Oligoarthritis	RF − Polyarthritis	RF + Polyarthritis	Psoriatic arthritis	Enthesitis-related arthritis	Undifferentiated arthritis	All JIA patients	Healthy
	*N* = 2	*N* = 56	*N* = 17	*N* = 13	*N* = 4	*N* = 7	*N* = 1	*N* = 100	*N* = 204
Female	1 (50%)	34 (60.7%)	11 (64.7%)	9 (69.2%)	3 (75%)	1 (14.3%)	0 (0%)	59 (59%)	106 (52%)
Age at visit	7.2 (6.2–8.2)	11 (7.9–15.4)	12.2 (10.9–16.9)	14.2 (12.2–15)	11.1 (9.6–12.6)	11 (6.2–12.2)	13.2 (13.2–13.2)	11.9 (9.2–15)	7.9 (4–9.9)^#^
Age at onset	4.4 (4.2–4.7)	7 (3.9–10.2)	8 (5–10)	10.2 (9.1–12.6)	9.6 (7.1–11.3)	4.6 (4.6–7.5)	9.4 (9.4–9.4)	7.6 (4.6–10.5)	
Disease duration	2.7 (1.5–4)	3.3 (2–5.9)	5.2 (2.5–7.3)	3.9 (0.7–5.2)	1.8 (1.3–2.5)	1.7 (1.6–6.6)	3.7 (3.7–3.7)	3.3 (1.9–5.9)	
ESR	30 (15–45)	7 (3–19)	15 (6–20)	32 (21–36)	7 (2–20)	6 (2–18)	12 (12–12)	12 (4–21)*	
MD VAS	8 (8–8)	4 (3–5)	4 (2–5)	7 (4–9)	4 (2–7)	3.5 (2.5–4.5)	(–)	4 (3–5)	
No. swollen joints	4 (4–4)	0 (0–1.5)	2 (0–8)	11 (8–12)	3 (1-4.5)	0 (0–2)	4 (4–4)	1 (0–4)^#^	
No. joints with pain	2.5 (0–5)	3 (2–4)	9 (5–13)	12 (9–15)	7 (4–10)	4 (4–5)	5 (5–5)	4 (2–8)^#^	
No. joints with LOM	4.5 (4–5)	0.5 (0–2)	5 (1–10)	12 (11–14)	6 (3–14)	3 (2–5)	16 (16–16)	2 (0–6)^#^	
No. active joints	4.5 (4–5)	1.5 (0–3.5)	5 (1–10)	12 (9–16)	4.5 (3–7)	3 (3–5)	8 (8–8)	3 (0–6)^#^	
Active systemic features	1 (50%)	2 (3.6%)	0 (0%)	1 (7.7%)	0 (0%)	0 (0%)	0 (0%)	4 (4%)^#^	
ANA status	0 (0%)	7 (12.5%)	2 (11.8%)	4 (30.8%)	0 (0%)	0 (0%)	1 (100%)	14 (14%)	
Uveitis	0 (0%)	5 (8.9%)	0 (0%)	1 (7.7%)	0 (0%)	1 (14.3%)	0 (0%)	7 (7%)	
PF total score	12.5 (0–25)	2 (0–3)	5 (3–8)	9 (2–16)	2 (1–4)	3 (0–5)	0 (0–0)	2.5 (0–5)*	0 (0–0)^#^
Pain VAS	5 (1–9)	3 (1.5–4.5)	4 (1-5.5)	5 (3–7)	4.5 (3.3–5.5)	3 (1.5–3.5)	0.5 (0.5–0.5)	3.5 (1.8-5)	0 (0–0)^#^
Disease activity VAS	4.8 (1–8.5)	2.8 (1-4.5)	4 (1.5-6)	6 (5–8)	3.3 (1.3–5.3)	3.5 (2.5-5)	0 (0–0)	3.5 (1.3–5.5)*	
Well-being VAS	5.8 (2.5-9)	3 (1-4.3)	3.5 (1-5.5)	6 (3.5-8)	3 (1.8–4.5)	3 (2–5)	0 (0–0)	3 (1.5-5)*	
HRQoL PhH	5 (2–8)	2 (1–4)	3 (2–6)	6 (2–7)	2.5 (1.5–4.5)	2 (1–4)	0 (0–0)	2 (1–4.5)	0 (0–5)^#^
HRQoL PsH	6.5 (4–9)	1 (0-3.5)	3 (1–6)	2 (0–4)	2 (0–7)	2 (0–5)	0 (0–0)	2 (0–5)	1 (0–3)
HRQoL total score	11.5 (6–17)	4 (1–7)	8 (3–11)	8 (4–10)	4.5 (1.5–11.5)	6 (1–6)	0 (0–0)	5 (2–8)	3 (0–99)
Pain/swell. in > 1 joint	1 (50%)	50 (89.3%)	15 (88.2%)	13 (100%)	4 (100%)	5 (71.4%)	0 (0%)	88 (88%)	0 (0%)^#^
Morning stiffness > 15 min	1 (50%)	17 (30.4%)	10 (58.8%)	11 (84.6%)	2 (50%)	2 (28.6%)	0 (0%)	43 (43%)	0 (0%)^#^
Subjective remission	2 (100%)	39 (69.6%)	13 (76.5%)	12 (92.3%)	3 (75%)	4 (57.1%)	0 (0%)	73 (73%)	
In treatment	2 (100%)	41 (73.2%)	15 (88.2%)	12 (92.3%)	4 (100%)	6 (85.7%)	0 (0%)	80 (80%)	
Reporting side effects	1 (50%)	4/41 (9.8%)	2/15 (13.3%)	3/12 (25%)	0 (0%)	1/6 (16.7%)	–	11/80 (13.8%)	
Taking medication regularly	2 (100%)	39/41 (95.1%)	15/15 (100%)	11/12 (91.7%)	4 (100%)	6/6 (100%)	–	77/80 (96.3%)	
With problems attending school	1/1 (100%)	4/34 (11.8%)	1/12 (8.3%)	0 (0%)	1/3 (33.3%)	1/5 (20%)	0 (0%)	8/60 (13.3%)	0 (0%)
Satisfied with disease outcome	1 (50%)	42 (75%)	8 (47.1%)	3 (23.1%)	3 (75%)	5 (71.4%)	1 (100%)	63 (63%)	

Data related to the JAMAR refers to the 100 JIA patients and to the 105 healthy subjects for whom the questionnaire has been completed by the parents

JAMAR, Juvenile Arthritis Multidimensional Assessment Report; ESR, erythrocyte sedimentation rate; MD, medical doctor; VAS, visual analogue scale (score 0–10; 0 = no activity, 10 = maximum activity); LOM, limitation of motion; ANA, anti-nuclear antibodies; PF, physical function (total score ranges from 0 to 45); HRQoL, health-related quality of life (total score ranges from 0 to 30); PhH, physical health (total score ranges from 0 to 15); PsH, psychosocial health (total score ranges from 0 to 15)

*p* values refers to the comparison of the different JIA categories or to JIA versus healthy. *p < 0.05, **p < 0.001, ^#^*p* < 0.0001

A total of 205/304 (67.4%) subjects had the parent version of the JAMAR completed by a parent (100 from parents of JIA patients and 105 from parents of healthy children). The JAMAR was completed by 185/205 (90.2%) mothers and 20/205 (9.8%) fathers. The child version of the JAMAR was completed by 170/304 (55.9%) children age 7.9 or older.

### Discriminant validity

The JAMAR results are presented in Table 1, including the scores (median (1st–3rd quartile)) obtained for the PF, the PhH, the PsH subscales and total score of the HRQoL scales. The JAMAR components discriminated well between healthy subjects and JIA patients.

In summary, the JAMAR revealed that JIA patients had a greater level of disability and pain, as well as a lower HRQoL than their healthy peers. However, there was no significant difference between healthy subjects and their affected peers in school-related item and in PsH subscale and in the total score of HRQoL.

### Psychometric issues

The main psychometric properties of both parent and child versions of the JAMAR are reported in Table 2. The following “Results” section refers mainly to the parent’s version findings, unless otherwise specified.

**Table 2:** Main psychometric characteristics between the parent and child version of the JAMAR

	Parent *N* = 100/205	Child *N* = 71/170
Missing values (1st–3rd quartiles)	No missing values	No missing values
Response pattern	PF and HRQoL positively skewed	PF and HRQoL positively skewed
Floor effect, median		
PF	85.0%	80.3%
HRQoL PhH	58.0%	59.2%
HRQoL PsH	62.0%	74.6%
Pain VAS	5.0%	5.6%
Disease activity VAS	13.0%	14.1%
Well-being VAS	9.0%	11.3%
Ceiling effect, median		
PF	0.0%	1.4%
HRQoL PhH	2.0%	2.8%
HRQoL PsH	1.0%	1.4%
Pain VAS	1.0%	0.0%
Disease activity VAS	0.0%	0.0%
Well-being VAS	0.0%	0.0%
Items with equivalent item-scale correlation	87% for PF, 80% for HRQoL	87% for PF, 100% for HRQoL
Items with items-scale correlation ≥ 0.4	87% for PF, 100% for HRQoL	100% for PF, 100% for HRQoL
Cronbach’s alpha		
PF-LL	0.81	0.85
PF-HW	0.93	0.94
PF-US	0.78	0.93
HRQoL-PhH	0.83	0.85
HRQoL-PsH	0.89	0.92
Items with item-scale correlation lower than the Cronbach alpha	93% for PF, 100% for HRQoL	100% for PF, 100% for HRQoL
Test–retest intraclass correlation		
PF total score	1.0	1.0
HRQoL-PhH	1.0	1.0
HRQoL-PsH	1.0	1.0
Spearman correlation with JIA core set variables, median		
PF	0.3	0.5
HRQoL PhH	0.3	0.4
HRQoL PsH	0.2	0.4
Pain VAS	0.4	0.5
Disease activity VAS	0.4	0.5
Well-being VAS	0.4	0.5

*JAMAR* Juvenile Arthritis Multidimensional Assessment Report, *JIA* juvenile idiopathic arthritis, *VAS* visual analogue scale, *PF* physical function, *HRQoL* health-related quality of life; *PhH*: physical health, *PsH* psychosocial health, *PF-LL* PF-lower limbs, *PF-HW* PF-hand and wrist, *PF-US* PF-upper segment

### Descriptive statistics (first Likert assumption)

There were no missing results for all JAMAR items, since data were collected through a web-based system that did not allow to skip answers and input null values. The response pattern for both PF and HRQoL was positively skewed toward normal functional ability and normal HRQoL. All response choices were used for the different HRQoL items except for items 8 and 10, whereas a reduced number of response choices was used for all the PF items except for items 1, 3, 4, 5, 8 and 10.

The mean ± SD of the items within a scale were roughly equivalent for the PF (except for item 5) and for the HRQoL items (except for items 3 and 5) (data not shown). The median number of items marked as not applicable was 1.0% (1–1.0%) for the PF and 1.0% (1–2.0%) for the HRQoL.

### Floor and ceiling effect

The median floor effect was 85.0% (65–93%) for the PF items, 58% (33–63%) for the HRQoL PhH items, and 62% (54–68%) for the HRQoL PsH items. The median ceiling effect was 0% (0–1%) for the PF items, 2% (1–2%) for the HRQoL PhH items, and 1% (0–1%) for the HRQoL PsH items. The median floor effect was 5% for the pain VAS, 13% for the disease activity VAS and 9% for the well-being VAS. The median ceiling effect was 1% for the pain VAS, 0% for the disease activity VAS and 0% for the well-being VAS.

### Equal items-scale correlations (second Likert assumption)

Pearson items-scale correlations corrected for overlap were roughly equivalent for items within a scale for 87% of the PF items, with the exception of PF items 5 and 15, and for 80% of the HRQoL items, with the exception of items 5 and 10.

### Items internal consistency (third Likert assumption)

Pearson items-scale correlations were ≥ 0.4 for 87% of items of the PF (except for PF items 5 and 15) and 100% of items of the HRQoL.

### Cronbach’s alpha internal consistency

Cronbach’s alpha was 0.81 for PF-LL, 0.93 for PF-HW, 0.78 for PF-US. Cronbach’s alpha was 0.83 for HRQoL-PhH and 0.89 for HRQoL-PsH.

### Interscale correlation

The Pearson correlation of each item of the PF and the HRQoL with all items included in the remaining scales of the questionnaires was lower than the Cronbach’s alpha, except for the PF item 13.

### Test–retest reliability

Reliability was assessed in 10 JIA patients, by re-administering both versions (parent and child) of JAMAR after a median of 7 days (7–7 days). The intraclass correlation coefficients (ICC) for the PF total score showed an almost perfect reproducibility (ICC = 1.0). The ICC for the HRQoL PhH and for the HRQoL PsH showed an almost perfect reproducibility (ICC = 1.0 for both).

### Convergent validity

The Spearman correlation of the PF total score with the JIA core set of outcome variables ranged from 0.3 to 0.7 (median = 0.3). The PF total score best correlation was observed with the disease activity VAS (*r* = 0.8, *p* < 0.001). For the HRQoL, the median correlation of the PhH with the JIA core set of outcome variables ranged from 0.2 to 0.5 (median = 0.3), whereas PsH ranged from 0.04 to 0.3 (median = 0.2). The PhH showed the best correlation with the parent’s assessment of pain (*r* = 0.6, *p* < 0.001) and the PsH showed the best correlation with the parent’s assessment of well-being (*r* = 0.3, *p* = 0.0004). The median correlations between the pain VAS, the well-being VAS, and the disease activity VAS and the physician-centred and laboratory measures were 0.4 (0.4–0.5), 0.4 (0.4–0.5), 0.4 (0.4–0.5), respectively.

## Discussion

In this study, the Latvian version of the JAMAR was cross-culturally adapted from the original standard English version with 2 forward and 2 backward translations. According to the results of the validation analysis, the Latvian parent and patient versions of the JAMAR possess satisfactory psychometric properties. The disease-specific components of the questionnaire discriminated well between patients with JIA and healthy controls. Notably, there was no significant difference between the healthy subjects and their affected peers in the psychosocial quality of life and in the total score of HRQoL and school-related problems. These findings indicates that children with JIA adapt well to the consequences of JIA, and have school performances comparable to those of their healthy peers. The functional ability questionnaire PF revealed to be able to discriminate between the different JIA subtypes with the children diagnosed with systemic arthritis having a higher degree of disability.

Psychometric evaluation was good for all domains with few exceptions: 2 PF items (bend down and bite a sandwich or an apple) showed a lower items internal consistency. However, the overall internal consistency was good for all the domains.

In the external validity evaluation, the Spearman’s correlations of the PF and HRQoL scores with JIA core set parameters ranged from weak to moderate.

The results obtained for the parent version of the JAMAR are very similar to those obtained for the child version, which suggests that children are equally reliable proxy reporters of their disease and health status as their parents.

The JAMAR is aimed to evaluate the side effects of medications and school attendance, which are other dimensions of daily life that were not previously considered by other HRQoL tools. This may provide useful information for intervention and follow-up in health care.

In conclusion, the Latvian version of the JAMAR was found to have satisfactory psychometric properties and it is, thus, a reliable and valid tool for the multidimensional assessment of children with JIA.

## References

[1] Filocamo G, Consolaro A, Schiappapietra B, Dalpra S, Lattanzi B, Magni-Manzoni S (2011). A new approach to clinical care of juvenile idiopathic arthritis: the Juvenile Arthritis Multidimensional Assessment Report. J Rheumatol.

[2] Ruperto N, Martini A (2011). Networking in paediatrics: the example of the paediatric rheumatology international trials organisation (PRINTO). Arch Dis Child.

[3] Consolaro A, Ruperto N, Filocamo G, Lanni S, Bracciolini G, Garrone M (2012). Seeking insights into the epidemiology, treatment and outcome of childhood arthritis through a multinational collaborative effort: introduction of the EPOCA study. Pediatr Rheumatol Online J.

[4] Bovis F, Consolaro A, Pistorio A, Garrone M, Scala S, Patrone E (2018). Cross-cultural adaptation and psychometric evaluation of the Juvenile Arthritis Multidimensional Assessment Report (JAMAR) in 54 languages across 52 countries: review of the general methodology. Rheumatol Int.

[5] Petty RE, Southwood TR, Baum J, Bhettay E, Glass DN, Manners P (1998). Revision of the proposed classification criteria for juvenile idiopathic arthritis: Durban, 1997. J Rheumatol.

[6] Petty RE, Southwood TR, Manners P, Baum J, Glass DN, Goldenberg J (2004). International League of associations for rheumatology classification of juvenile idiopathic arthritis: second revision, Edmonton, 2001. J Rheumatol.

[7] Filocamo G, Sztajnbok F, Cespedes-Cruz A, Magni-Manzoni S, Pistorio A, Viola S (2007). Development and validation of a new short and simple measure of physical function for juvenile idiopathic arthritis. Arthritis Rheum.

[8] Lovell DJ, Howe S, Shear E, Hartner S, McGirr G, Schulte M (1989). Development of a disability measurement tool for juvenile rheumatoid arthritis. The juvenile arthritis functional assessment scale. Arthritis Rheum.

[9] Howe S, Levinson J, Shear E, Hartner S, McGirr G, Schulte M (1991). Development of a disability measurement tool for juvenile rheumatoid arthritis. The juvenile arthritis functional assessment report for children and their parents. Arthritis Rheum.

[10] Singh G, Athreya BH, Fries JF, Goldsmith DP (1994). Measurement of health status in children with juvenile rheumatoid arthritis. Arthritis Rheum.

[11] Filocamo G, Davi S, Pistorio A, Bertamino M, Ruperto N, Lattanzi B (2010). Evaluation of 21-numbered circle and 10-centimeter horizontal line visual analog scales for physician and parent subjective ratings in juvenile idiopathic arthritis. J Rheumatol.

[12] Duffy CM, Arsenault L, Duffy KN, Paquin JD, Strawczynski H (1997). The juvenile arthritis quality of life questionnaire–development of a new responsive index for juvenile rheumatoid arthritis and juvenile spondyloarthritides. J Rheumatol.

[13] Varni JW, Seid M, Knight TS, Burwinkle T, Brown J, Szer IS (2002). The PedsQL(TM) in pediatric rheumatology—reliability, validity, and responsiveness of the pediatric quality of life inventory(TM) generic core scales and rheumatology module. Arthritis Rheum.

[14] Landgraf JM, Abetz L, Ware JE (1996). The CHQ user’s manual.

[15] Filocamo G, Consolaro A, Schiappapietra B, Ruperto N, Pistorio A, Solari N (2012). Parent and child acceptable symptom state in juvenile idiopathic arthritis. J Rheumatol.

[16] Nunnally JC (1978). Psychometric theory.

[17] Giannini EH, Ruperto N, Ravelli A, Lovell DJ, Felson DT, Martini A (1997). Preliminary definition of improvement in juvenile arthritis. Arthritis Rheum.

[18] Ware JE, Harris WJ, Gandek B, Rogers BW, Reese PR (1997). MAP-R for Windows: multitrait/multi-item analysis program—revised user’s guide. Version 1.0 ed.

